# Tension Pneumothorax During Rigid Bronchoscopy for Chronic Foreign Body Removal in a Child: A Case Report

**DOI:** 10.7759/cureus.5628

**Published:** 2019-09-11

**Authors:** Akshay Kumar, Nimisha Shiwalkar, Hunain Aslam, Purnadeo Persaud

**Affiliations:** 1 Cardiothoracic Surgery, Lokmanya Tilak Municipal Medical College & General Hospital, Mumbai, IND; 2 Anesthesiology, Lokmanya Tilak Municipal Medical College & General Hospital, Mumbai, IND; 3 Surgery, University of Missouri, Columbia, USA; 4 Kansas City University of Medicine and Biosciences, Kansas City, USA

**Keywords:** respiratory, resuscitation, imaging, intensive care, tension pneumothorax, tracheobronchial foreign body, rigid bronchoscopy, intercostal tube

## Abstract

Rigid bronchoscopy for chronic foreign body removal can cause rare but life-threatening complications in the form of tension pneumothorax. A two-year-old child who developed sudden cardiac arrest during the procedure required urgent chest tube insertion. Integrated team effort with effective communication prevented devastating neurological sequelae from hypoxic ischemic encephalopathy.

## Introduction

Bronchoscopy was developed and first used in 1895 for removing foreign bodies from the main stem bronchi, when Gustav Killian removed a piece of bone from the right main-stem bronchus of a 36-year-old man [1]. Performing these procedures in airway of pediatric patients poses a special challenge to both the anesthesiologist and the surgeon. Pneumothorax remains a rare but devastating complication instigating from lower airway manipulation during rigid bronchoscopy [2]. Rothmana and Boeckman reported that the probability of developing pneumothorax during rigid bronchoscopy for the removal of foreign body is approximately one in 100 cases [3]. The possibility of tension pneumothorax should be borne in mind in a mechanically ventilated patient with persistently rising airway pressures and progressively diminishing cardiac output. We herein report a challenging case of foreign body removal, where the patient developed cardiac arrest due to intraoperative pneumothorax and was revived with continued successful cardiopulmonary resuscitation. In the particular case of the patient being a minor, a written informed parental consent was taken to publish this case report.

## Case presentation

**Figure 1:**
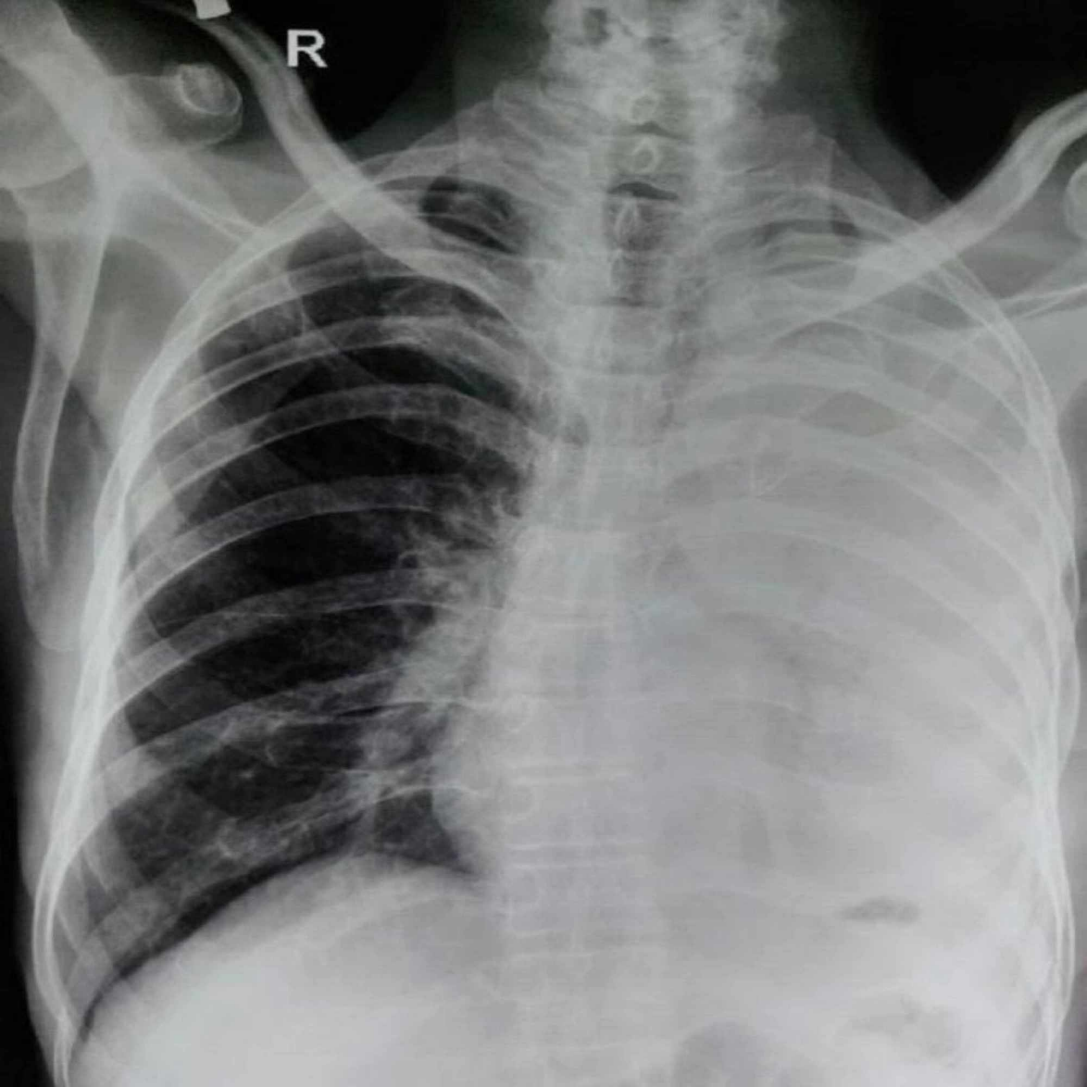
Chest X-ray showing haziness in the left chest and emphysematous changes in the right side of the chest

A two-year-old male child, weighing 15 kg, was presented to the outpatient clinic with complaints of recurrent lower respiratory tract infections for one year. Air entry was decreased in the left lower zone. Rest of respiratory as well as cardiac and neurological examinations were within normal limits. Chest X-ray revealed emphysematous changes on the right side and shift of mediastinum to the left (Figure 1).

**Figure 2:**
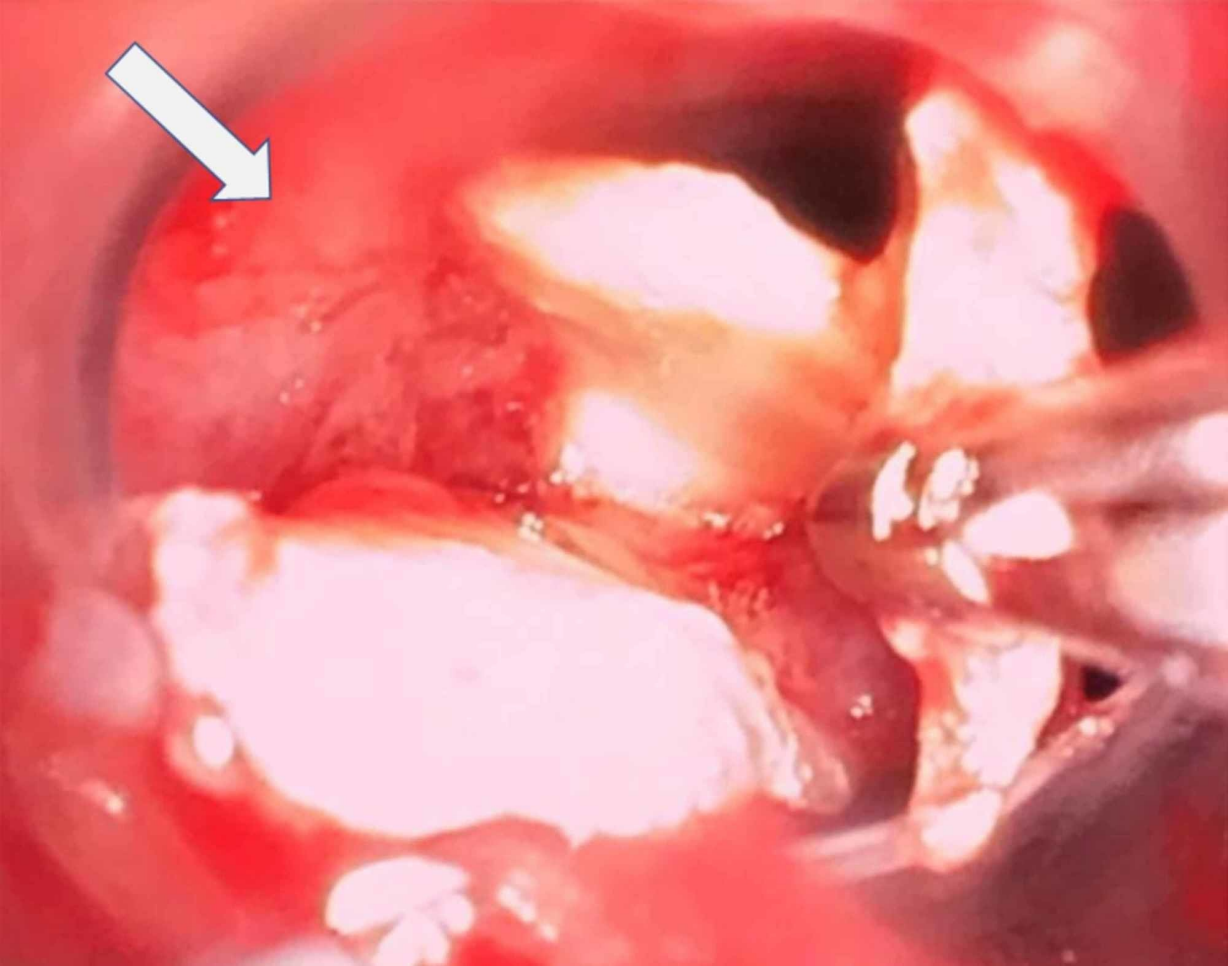
Intraoperative picture of bronchoscopy showing foreign body being retrieved by grasper

Contrast-enhanced CT scan revealed focal stenosis of left lower lobe bronchus due to possible foreign body or bronchial stricture, and further evaluation with bronchoscopy was recommended. Thus, the patient was taken up for rigid bronchoscopic evaluation for removal of foreign body under general anesthesia with controlled manual ventilation. The patient was premedicated with intravenous Inj glycopyrrolate 0.06 mg, Inj midazolam 0.3 mg, Inj ranitidine 15 mg, Inj ondansetron 1.5 mg, and Inj fentanyl 30 µg. The patient was then induced with Inj propofol in graded doses till loss of consciousness followed by Inj suxamethonium 1 mg/kg. Rigid bronchoscope was then introduced, and anesthesia was maintained through the side port of the bronchoscope with 100% O_2_, sevoflurane, and intermittent atracurium. Bronchoscopy revealed foreign body in the left lower bronchus at its take off from the left main stem bronchus. Subsequently, multiple attempts were made to remove the foreign body using graspers (Figure 2). 

**Figure 3:**
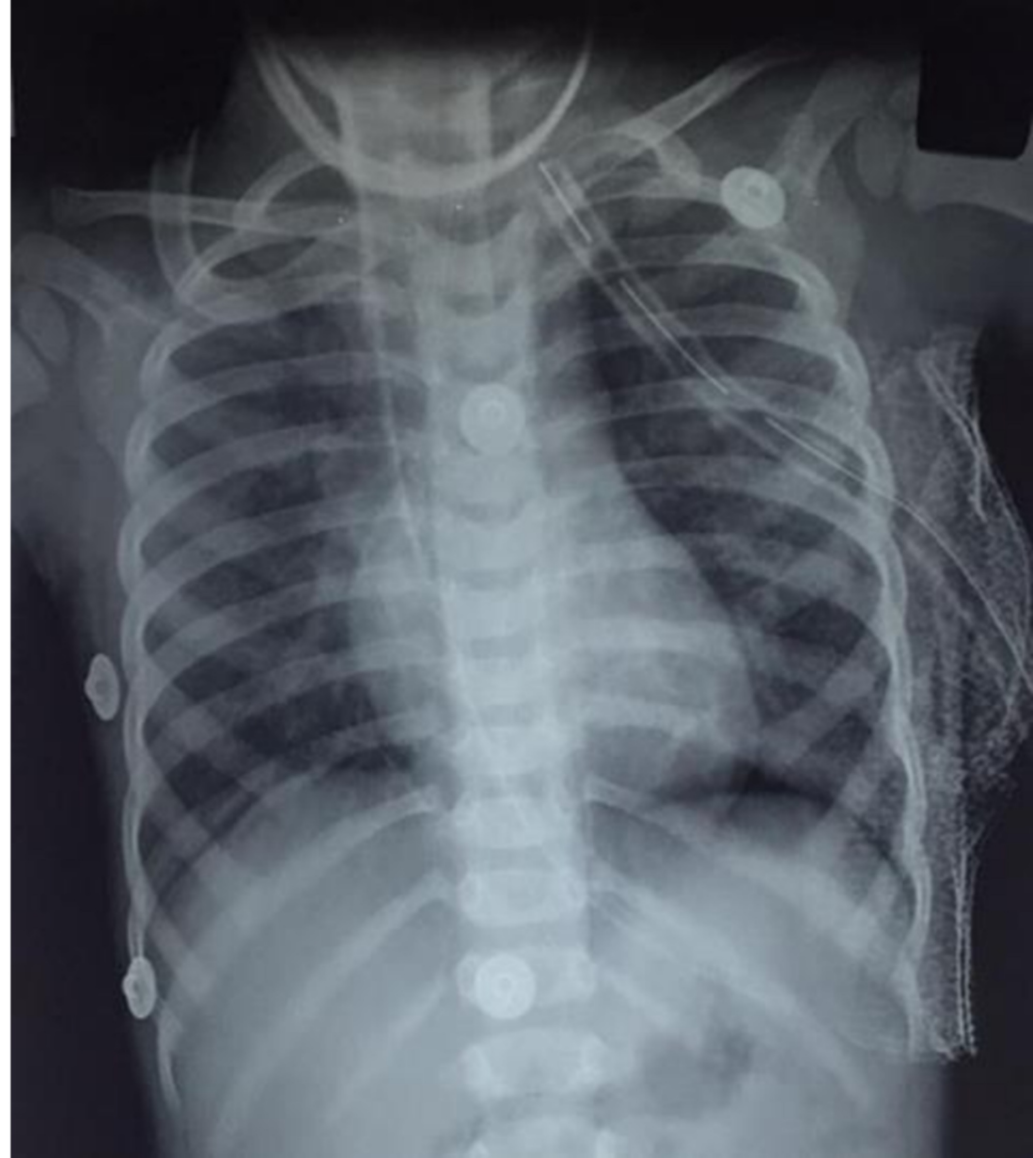
Intraoperative chest X-ray after insertion of intercostal drainage tube

After about 30 minutes into the procedure, the patient suddenly developed bradycardia and dropped his saturations to low 70s culminating in asystole. Cardiopulmonary resuscitation (CPR) was initiated immediately as per the PALS (Pediatric Advanced Life Support) protocol. Simultaneously, a search for cause for cardiac arrest revealed pneumothorax on the left side. Immediately intercostal drainage was inserted in left chest in the fifth intercostal space in the anterior axillary line (Figure 3).

The child was shifted to the intensive care unit in hemodynamically stable (hazard ratio-104/min, blood pressure-94/62 mmHg, SPO_2_-95%) but guarded condition. The hemodynamic profile of the child in the pre-, intra-, and postoperative period after resuscitation is shown in Table 1. On postoperative day, one patient woke up and did not show any signs of respiratory or neurological deficit and was extubated. There was no air leak or residual pneumothorax, and his chest tube was removed on day two. His postoperative recovery was uneventful and was discharged from hospital after few days.

**Table 1:** Hemodynamic profile of the child in the preoperative and intraoperative during rigid bronchoscopy, prior to arrest, post chest tube insertion, and postoperative in the ICU BP, blood pressure; HR, hazard ratio; ICU, intensive care unit.

	Preop	Intraop	Intraoperative (30 min)	Prior to Arrest	Post Chest tube Insertion	Postop	In ICU
Temp (Celsius)	37.4	37.4	37.5	37.2	37.0	37.8	37.4
HR (per minute)	94	98	64	40	140	118	104
BP (syst/diastolic) Mean (mmHg)	88/58 (68)	82/56 (55)	68/48 (62)	----	58/38 (50)	84/58 (68)	94/62 (104/84)
SpO_2_	99	99	72	34	94	92	95

## Discussion

Rigid bronchoscopy for retrieval of foreign bodies is a common procedure in pediatric age group, though it is a very challenging case scenario. Complications include bleeding, infection, laryngeal edema, laryngospasm, bronchospasm, hypoxemia, airway injury with tracheobronchial laceration, and pneumothorax usually resulting from direct trauma to tracheobronchial tree [4,5]. Though the possibility of pneumothorax is less than one percent, it remains one of the dreaded complications during bronchoscopy [6]. The addition of positive pressure due to high-frequency jet ventilation converts pneumothorax into a tension pneumothorax. This occurs when one-way valve mechanism develops because of tracheo-bronchial instrumentation leading inadvertent rent in airway, and thereby allowing the air to enter the pleural cavity during positive pressure ventilation and preventing exit during expiratory phase. As the air builds up in the pleural space, ipsilateral lung is compressed followed by mediastinal shift and compression of contralateral lung and intrathoracic vasculature leading to severe hypoxemia and cardiovascular compromise [7]. The development of tension is dependent on a pressure gradient between intrapleural pressure (IPP) and alveolar pressure. Continued ventilation increases gas flow through the pleural defect, allowing more air to pass per unit time. This accentuates the rise in IPP with earlier mechanical compressive effects and rapid progress to cardiorespiratory collapse [8-10]. Early and prompt diagnosis of pneumothorax during anesthesia, however, can be tricky as the symptoms may be masked due to anesthesia in a mechanically ventilated patient [11]. Diagnosis in these patients rests upon high index of suspicion and recognition of supportive diagnostic features. A sudden fall in SP0_2_ ensues followed by hypotension (over a few minutes) as was noted in Steier’s large case series and is consistently found in the individual case reports [12]. Progression to tension pneumothorax can lead to a rapid increase in airway pressures culminating in bradycardia and cardio respiratory arrest. Because of this sudden nature of presentation, tension pneumothorax is more often than likely to be missed in ventilated patients [13]. A low threshold for performing tube or finger thoracostomy must be maintained in mechanically ventilated patients [14]. This reiterates that continuous vigilant monitoring is essential to alert the anesthesiologist of a worsening situation.Hussain et al. reported a one-year-old female child developing tension pneumothorax following rigid bronchoscopy for acute foreign body aspiration [15]. In more grievous injury to the tracheo-bronchial tree requiring surgical repair or airway reconstruction, it is prudent to place the child on extracorporeal circulatory support, repair the injury, and provide lung protective ventilation [16]. To the best of our knowledge, this is the first case reported in the literature for tension pneumothorax complicated by cardiac arrest following rigid bronchoscopy for chronic foreign body removal in such a small child managed successfully.

## Conclusions

A high index of suspicion must be maintained for pneumothorax during rigid bronchoscopy whenever sudden deterioration of hemodynamic or hypoxia ensues. Intraoperative catastrophe from which a child cannot be revived or suffers irreversible neurological damage can hamper the morale of the healthcare team. An integrated teamwork emphasizing on efficient communication and coordination between the surgeon, anesthesiologist, scrub nurse, circulating nurse, technicians, and perfusionist is of utmost importance in saving the child’s life.
